# Supplementary material for: HIVprotI: an integrated web based platform for prediction and design of HIV proteins inhibitors
Source: J Cheminform. 2018 Mar 9;10:12. doi: 10.1186/s13321-018-0266-y (PMC5845081; doi:10.1186/s13321-018-0266-y)
Supplement: Supplementary file 2 — Additional file 2. Source code of HIVProtI web server. [file 13321_2018_266_MOESM2_ESM.zip › HIVprotI_Source-code/hivprotI/submit_analog.php]

php include("head.php");
$ran = $\_REQUEST['ran'];
#$email=$\_REQUEST['email'];
?
php
$dir="/home/gpsr/webserver/cgidocs/tmp/manojk/hivproti/first\_$ran";
if (file\_exists("$dir/cat\_final\_accuracy"))
{
shell\_exec("perl for\_graph.pl $ran");
echo"<br  
**Predicted percent inhibition of different analogs on selected proteins**
  
  
";
include("/home/gpsr/webserver/cgidocs/tmp/manojk/hivproti/first\_$ran/cat\_final\_accuracy");
$x=file\_get\_contents("$dir/graph\_final");
$x=str\_replace("][","], [",$x);
##### Download #########
$xy=file\_get\_contents("$dir/cat\_final\_accuracy\_download");
#$x=str\_replace("][","], [",$x);
#$xx=str\_replace("],[","]\n[",$x);
#$xxx=str\_replace("]"," ",$xx);
#$xxxx=str\_replace("["," ",$xxx);
$FP6=fopen("$dir/analogs\_result.txt","w");
fwrite($FP6,"$xy");
fclose($FP6);
echo "  
";
#######################
########Plot Graph#########
echo"

|  |  |
| --- | --- |
| "; /\*echo " |";\*/
echo "  |

";
/\*\*
###########################
##### email sending####
$to = $email;
$subject = 'AVCpred results';
$bound\_text = "AVCpred results";
$bound = "--".$bound\_text."\r\n";
$bound\_last = "--".$bound\_text."--\r\n";
$headers = "From: manojk@imtech.res.in \r\n";
$headers .= "Reply-To: manojk@imtech.res.in \r\n";
$headers .= "MIME-Version: 1.0\r\n"
."Content-Type: multipart/mixed; boundary=\"$bound\_text\"";
$message .= "If you can see this MIME than your client doesn't accept MIME types!\r\n"
.$bound;
$message .= "Content-Type: text/html; charset=\"iso-8859-1\"\r\n"
."Content-Transfer-Encoding: 7bit\r\n\r\n"
."Dear User\n  
 Thanks for using AVCpred. Result of your query is available from URL http://crdd.osdd.net/servers/avcpred/submit\_analog.php?ran=$ran&email=$email , your result will be available for next five days.  
  
 \n\r With Regards \n\r   
  
Manoj Kumar \n\r   
  
URL: http://www.imtech.res.in/manojk/ \n\r";
if($email!=""){mail($to, $subject, $message, $headers);}
#######################
\*\*/
#exec("rm -r $dir/\*\_acc $dir/\*\_list $dir/\*.x $dir/graph.\* $dir/desc\* $dir/bracket $dir/\*.q $dir/cells $dir/header $dir/ic $dir/ids $dir/ids\_mod $dir/list $dir/list\_sdf $dir/model\_req $dir/temp $dir/tlist $dir/tmp $dir/topkey $dir/analog $dir/mols $dir/outtmp $dir/raw $dir/strctr $dir/test1");
}
else
{
//echo"$email  
";
echo"  
  
  
  
  
  
";
if($email==""){
echo "

#### Please wait.... (2-5 min)

";
echo "";}
else{
echo "

#### Please wait.... (2-5 min)

";
echo "";}
}
?>
php include("foot.php")?
